# General practitioner reported incidence of Lyme carditis in the Netherlands

**DOI:** 10.1007/s12471-015-0744-z

**Published:** 2015-09-09

**Authors:** A. Hofhuis, S.M. Arend, C.J. Davids, R. Tukkie, W. van Pelt

**Affiliations:** 1 Centre for Infectious Disease Control Netherlands, National Institute for Public Health and the Environment (RIVM), Bilthoven, The Netherlands; 2 Department of Infectious Diseases, Leiden University Medical Centre, Leiden, The Netherlands; 3 Department of Cardiology, Kennemer Gasthuis, Haarlem, The Netherlands

**Keywords:** Lyme carditis, Lyme borreliosis, Incidence, Medical diagnoses, Medical record review

## Abstract

**Background:**

Between 1994 and 2009, incidence rates of general practitioner (GP) consultations for tick bites and erythema migrans, the most common early manifestation of Lyme borreliosis, have increased substantially in the Netherlands. The current article aims to estimate and validate the incidence of GP-reported Lyme carditis in the Netherlands.

**Methods:**

We sent a questionnaire to all GPs in the Netherlands on clinical diagnoses of Lyme borreliosis in 2009 and 2010. To validate and adjust the obtained incidence rate, medical records of cases of Lyme carditis reported by GPs in this incidence survey were reviewed and categorised according to likelihood of the diagnosis of Lyme carditis.

**Results:**

Lyme carditis occurred in 0.2 % of all patients with GP-reported Lyme borreliosis. The adjusted annual incidence was six GP-reported cases of Lyme carditis per 10 million inhabitants, i.e. approximately ten cases per year in 2009 and 2010.

**Conclusions:**

We report the first incidence estimate for Lyme carditis in the Netherlands, validated by a systematic review of the medical records. Although Lyme carditis is an uncommon manifestation of Lyme borreliosis, physicians need to be aware of this diagnosis, in particular in countries where the incidence of Lyme borreliosis has increased during the past decades.

## Introduction

Lyme borreliosis is an infectious disease caused by *Borrelia burgdorferi* sensu lato species, and transmitted through tick bites. In Europe, the main vector of Lyme borreliosis is *Ixodes ricinus,* and at least five *Borrelia burgdorferi* sensu lato species can cause Lyme borreliosis: *B. afzelii* and *B. garinii*, and to a lesser degree also *B. burgdorferi* sensu stricto, *B. bavariensis* and *B. spielmanii*. Erythema migrans is the most common early manifestation of Lyme borreliosis, while disseminated infection can develop two to three months after initial infection, manifesting in the nervous system, joints, skin and sometimes the heart [1]. Symptomatic cardiac involvement associated with Lyme borreliosis usually occurs simultaneously with or shortly after other disease manifestations of Lyme borreliosis. Lyme carditis typically manifests as conduction abnormalities with varying degrees of atrioventricular conduction defects (AV block), although other cardiac manifestations such as endomyocarditis and pericarditis have also been reported [1, 2]. Although usually self-limiting, these cardiac conduction abnormalities can occasionally be life-threatening, as illustrated by a recent publication on three sudden death cardiac events associated with Lyme carditis in the United States [3]. The diagnosis of Lyme carditis is usually based on acute onset of relevant symptomatic cardiac disorders, with electrocardiogram (ECG) abnormalities and positive serology for *Borrelia* antibodies, and can be supported by a history of Lyme borreliosis or tick bite. Alternative explanations for the cardiac condition need to be excluded [1, 2].

Nationwide cross-sectional retrospective surveys among all general practitioners (GPs) in the Netherlands have shown a persistent increase in the incidence of GP consultations for tick bites and erythema migrans between 1994 and 2009, with up to 93,000 cases with tick bites and 22,000 cases with erythema migrans seen by all GPs in the Netherlands in 2009 [4]. Neighbouring countries have also reported an increasing incidence of Lyme borreliosis [5]. To estimate the incidence of Lyme carditis in the Netherlands in 2009 and 2010, we surveyed all GPs in the Netherlands, as part of a broader survey on physician-reported incidence of all Lyme borreliosis manifestations [6]. To verify and adjust the crude (GP reported) incidence rate of Lyme carditis from this nationwide cross-sectional retrospective incidence survey, we reviewed the medical records of Lyme carditis cases reported by GPs.

## Methods

To rapidly assess a nationwide representative incidence rate for Lyme carditis, we performed a two-step approach. Firstly, a broad inquiry was required to detect a very uncommon disease manifestation such as Lyme carditis. We sent a two-page retrospective questionnaire about Lyme borreliosis diagnoses during the two-year period of 2009–2010, also including diagnoses of Lyme carditis, to all (*n* = 9178) GPs in the Netherlands. In the Netherlands, Lyme carditis is usually diagnosed and treated by medical specialists in the hospital, and not by GPs. However, in general, patients are required to consult a GP to be referred to a hospital, except in urgent situations, and medical specialists report back to the GP. As every person in the Netherlands is registered with only one GP, the practice populations of reporting GPs were used to calculate crude annual incidence rates and national estimates of annual total numbers among the 16.6 million population of the Netherlands in 2010. The accompanying clinical case definition with regard to Lyme carditis was: ‘Acute onset of atrioventricular (I–III) conduction defects, rhythm disturbances, sometimes myocarditis or pancarditis. Alternative explanations must be excluded’, as proposed by Stanek et al. [1]. To achieve a high GP-response rate, we inquired about clinical diagnoses of Lyme carditis in the first step of our two-step approach, not asking the GP to look into laboratory diagnostics that had been ordered and interpreted by medical specialists in the hospital. Instead, the laboratory diagnostics were verified in the second part of our two-step approach: systematic review of medical records of the GP-reported Lyme carditis patients, to verify this crude incidence rate of Lyme carditis, obtained from our larger incidence survey on all manifestations of Lyme borreliosis [6]. We asked cooperation of all GPs who reported Lyme carditis diagnoses in the questionnaire. All relevant data for the systematic medical record review were collected by one medical master student (co-author CD), using a case record form (CRF). Assessed variables included: medical history, clinical symptoms (such as palpitations, dizziness, dyspnoea, oedema, orthopnoea, syncope, chest pain, fatigue), cardiac abnormalities visible on ECG, use of cardio-active medications, blood chemistry, *Borrelia*-specific tests such as ELISA, immunoblots, PCR and culture results, treatment and clinical course.

Considering the complexities in the diagnosis of Lyme carditis, the collected medical records were categorised according to the likelihood of diagnosis, into ‘very likely’, ‘likely’, ‘possible’, and ‘not Lyme carditis’ (Table 1). In the absence of validated and standardised criteria, we designed our own criteria for this purpose, based on cardiac symptoms, ECG abnormalities consistent with Lyme carditis (any degree of atrioventricular block, or any other type of conduction disturbance), positive serology for *Borrelia*, anamnestic tick bites or Lyme borreliosis, clinical recovery after antibiotic treatment, and recovery of the ECG abnormality. Based on the information available in the medical records, other possible causes for the cardiac symptoms were excluded, among which syphilis, thyroid disorders, familial arrhythmias, arrhythmogenic drugs, and ischaemia. We adjusted the crude incidence rate from this incidence survey according to the proportion of GP-reported Lyme carditis that we categorised as very likely or likely diagnosis. Additionally, to correct for ‘telescoping bias’ [7], the incidence rate was adjusted by the proportion of GP-reported Lyme carditis cases with a clinical presentation within the targeted period of 2009 and 2010.

**Table 1:** Criteria for classification of medical records according to likelihood of the diagnosis Lyme carditis

**Very likely**	Acute onset of cardiac symptoms^a^
	+ ECG abnormality consistent with Lyme carditis^b^
	+ positive serology ^c^ for *Borrelia*
Or:	Acute onset of cardiac symptoms^a^
	+ no ECG abnormality consistent with Lyme carditis ^b^/no ECG performed
	+ positive serology^c^ for *Borrelia*
	+ anamnestic tick bite/anamnestic erythema migrans/diagnosed Lyme arthritis/
	diagnosed Lyme neuroborreliosis
	+ clinical recovery after antibiotic treatment
**Likely**	Acute onset of cardiac symptoms^a^
	+ positive serology^c^ for *Borrelia*
	+ clinical recovery after antibiotic treatment
Or:	ECG abnormality consistent with Lyme carditis^b^
	+ positive serology^c^ for *Borrelia*
Or:	Acute onset of cardiac symptoms^a^
	+ ECG abnormality consistent with Lyme carditis^b^
	+ *Borrelia* serology not performed
	+ anamnestic tick bite/anamnestic erythema migrans/diagnosed Lyme arthritis/
	diagnosed Lyme neuroborreliosis
	+ clinical recovery after antibiotic treatment/recovery ECG
**Possible**	Acute onset of cardiac symptoms^a^
	+ positive serology^c^ for *Borrelia*
	+ anamnestic tick bite/anamnestic erythema migrans/diagnosed Lyme arthritis/
	diagnosed Lyme neuroborreliosis
Or:	Acute onset of cardiac symptoms^a^
	+ positive serology^c^ for *Borrelia*
Or:	Acute onset of cardiac symptoms^a^
	+ *Borrelia* serology not performed
	+ anamnestic tick bite/anamnestic erythema migrans/diagnosed Lyme arthritis/
	diagnosed Lyme neuroborreliosis
**Not Lyme carditis**	Negative serology^c^ for *Borrelia*

Other plausible causes for the cardiac symptoms were excluded, among which syphilis, thyroid disorders, familial arrhythmias, arrhythmogenic drugs, and ischaemia.

Within each classification, criteria are ordered by their importance for the diagnosis of Lyme carditis.

^a^Cardiac symptoms such as palpitations, dizziness, dyspnoea, oedema, orthopnoea, syncope, chest pain, fatigue.

^b^Electrocardiogram (ECG) showing cardiac disorders consistent with Lyme carditis, such as any degree of atrioventricular block, or any other type of conduction disturbance.

^c^ELISA and/or IgG immunoblot and/or IgM immunoblot for *Borrelia* antibodies.

## Results

The response rate to the question on Lyme carditis (Table 2) in the questionnaire on all Lyme borreliosis manifestations was 33 % (3067/9178) among GPs, whose practice population includes about 7.7 million persons. This represents 46 % of the 16.6 million inhabitants of the Netherlands. Thirty-nine cases of Lyme carditis were reported by 38 GPs. These 39 GP reports of Lyme carditis per 7.7 million practice population during two years account for 0.2 % of 15,624 GP reports on all Lyme-related diagnoses (not validated) in our incidence survey on all manifestations of Lyme borreliosis [6]. Twenty-one GPs who together reported 22 cases answered our request for cooperation in the medical record review of the reported cases. Finally, eight medical records out of 39 GP reports could be reviewed (Table 2). We discovered three invalid reports, as two reports were withdrawn after the reporting GPs declared to have marked a Lyme carditis case on the questionnaire erroneously, and one case turned out to have been reported twice by two GPs working in partnership. Table 2 shows the data collection and classification of the eight reviewed Lyme carditis cases, according to likelihood of diagnosis. Table 3 provides an overview of the collected information from the reviewed medical records. Six out of eight reviewed medical records satisfied the criteria for a very likely diagnosis of Lyme carditis, one medical record was categorised as likely, and one was categorised as not Lyme carditis. With the information gained in this medical record review we calculated an adjusted incidence rate for very likely plus likely Lyme carditis, including the three invalid reports into the denominator of reviewed GP reports, resulting in 55 % (6/11) very likely diagnoses, and 9 % (1/11) likely diagnoses. Among the reviewed medical records, three out of eight cases (38 %) had a clinical presentation within the targeted period of 2009 and 2010. The adjusted annual incidence rate for very likely plus likely Lyme carditis diagnoses was 6 (95 % CI: 4–8) per 10 million inhabitants of the Netherlands in 2009 and 2010. This yields an estimated total number of ten very likely plus likely cases diagnosed nationwide per year in 2009 and 2010, of which nine very likely diagnoses.

**Table 2:** Data collection and classification of reviewed medical records, annual incidence of GP-reported Lyme carditis diagnoses per 10 million inhabitants and annual total numbers among the 16.6 million inhabitants of the Netherlands in 2009 and 2010

**Incidence questionnaire sent to GPs**	***N***	**(% of 9178)**
Response to question on Lyme carditis	3067	*(33.4 %)*
GP practice population	7,682,803	
Reports of diagnosed Lyme carditis	39	
**Annual incidence of Lyme carditis in 2009 and 2010 per 10 million inhabitants, based on 39 GP reports of Lyme carditis per 7,682,803 practice population**		
Crude incidence for Lyme carditis	25	(95 % CI: 18–34)/10,000,000
**Medical record review**	***N***	**(% of 22)**
Response on GP reported cases	22	
GP unable to recall identity	7	*(31.8 %)*
Unwilling to cooperate	4	*(18.2 %)*
Invalid reports of Lyme carditis	3	*(13.6 %)*
Medical records reviewed	8	*(36.4 %)*
**Classification of reviewed medical records**	***N***	**(% of 11)**
Very likely diagnosis	6	*(54.5 %)*
Likely diagnosis	1	*(9.1 %)*
Possible diagnosis	–	
Not Lyme carditis	1	*(9.1 %)*
Invalid report of Lyme carditis, not reviewed	3	*(27.3 %)*
**Clinical presentation within 2009 or 2010**	***N***	**(% of 8)**
	3	*(37.5 %)*
**Adjusted** ^a^ **annual incidence of Lyme carditis in 2009 and 2010 per 10 million inhabitants**		
Incidence for Lyme carditis	6	(95 % CI: 4–8)/10,000,000
National numbers	10	

*95 % CI:* 95 % confidence intervals.

^a^Adjusted for 63.6 % very likely + likely diagnoses, and for 37.5 % clinical presentations within the targeted period of 2009 or 2010.

**Table 3:** Characteristics of eight cases with Lyme carditis, collected through medical record review

Case#, Classification	Date clinical presentation	Gender & age	Cardiac symptoms (duration)	Relevant anamnesis	Serology & clinical chemistry	ECG, Chest X-ray	Treatment & clinical course
1, Very likely	July 2007	Male 52	Dyspnoea, angina pectoris (2–4 weeks)	Tick bite, EM (2–4 weeks)	ELISA IgG & IgM positive Immunoblot positive	ECG n.a. Chest X-ray: not performed	Clinical recovery, 2–4 weeks after 100 mg doxycycline b.i.d. for 30 days
2, Very likely	May 2010	Male 49	Palpitations, dyspnoea, syncope, angina pectoris, dizziness, fatigue (3 weeks)	EM, radiculitis (3 weeks)	ELISA positive Immunoblot positive. Intrathecal antibody response after 1st antibiotic treatment.	ECG: 1st degree AV-block Chest X-ray: not performed	No recovery after 100 mg doxycycline b.i.d. for 21 days Clinical and ECG recovery, 1 week after ceftriaxone 2 g q.d. IV for 14 days. Temporary pacemaker
3, Very likely	November 2005	Male 58	Dyspnoea, fatigue (1 month)	Flu-like symptoms (3 months)	ELISA IgG & IgM positive Immunoblot positive	ECG: ST-segment change (flat ST-wave inferolateral) Chest X-ray: not performed	No recovery after 100 mg doxycycline b.i.d. for 21 days No recovery after ceftriaxone 2 g q.d. IV for 14 days
4, Very likely	September 2009	Female 85	Palpitations, dyspnoea, orthopnoea/oedema, dizziness, fatigue (3 weeks)	EM (1 month)	ELISA IgG positive	ECG: 3rd degree AV-block Chest X-ray: cardiomegaly decompensatio cordis	After 100 mg doxycycline b.i.d. for 21 days, plus permanent pacemaker, diuretic, anticoagulant, Clinical recovery
5, Very likely	September 2005	Male 60	Dyspnoea, dizziness, fatigue (3 weeks)	Tick bite (3 weeks)	ELISA IgG positive Immunoblot positive	ECG: 3rd degree AV-block Chest X-ray: no abnormalities	Clinical and ECG recovery, 2 weeks after ceftriaxone 2gr q.d. IV for 14 days
6, Very likely	February 2011	Female 75	Dyspnoea, dizziness, fatigue (1–2 days)	Frequent exposure to tick bites, flu-like symptoms (duration n.a.)	ELISA IgM positive Immunoblot negative	ECG: 3rd degree AV-block Chest X-ray: no abnormalities	Clinical improvement and full ECG recovery, 2- 4 weeks after 100 mg doxycycline b.i.d. for 21 days, with permanent pacemaker, beta blocker, carbasalate calcium
7, Likely	November 2010	Male 70	Palpitations (2 weeks)	2007: Tick bite, EM Potential arrhythmogenic medication for present hypertension: beta blocker, calcium channel blocker	ELISA IgG positive Immunoblot positive	ECG no abnormalities Chest X-ray: not performed	Some temporary clinical improvement 2 weeks after 100 mg doxycycline b.i.d. for 14 days
8, Not Lyme carditis	December 2011	Male 70	Syncope, dizziness (1 year)	2005: LB with facial palsy, treated with ceftriaxone 2 g q.d. IV for 14 days Potential arrhythmogenic medication for hypertension, atrial fibrillation, mitral valve insufficiency: beta blocker	ELISA negative	ECG: no abnormalities Chest X-ray: cardiomegaly	Clinical recovery without antibiotic treatment. Start anticoagulant

*GP* general practitioner, *EM* erythema migrans, *LB* Lyme borreliosis, *ECG* electrocardiogram, *IV* intravenous, *q.d*. once per day, *b.i.d.* two times per day, *n.a.* not available.

## Discussion

We report the first incidence estimate of Lyme carditis in the Netherlands. Considering that not all patients with Lyme borreliosis are diagnosed correctly, we may have underestimated or overestimated its true occurrence. Misdiagnosis, misclassification and telescoping bias were reduced through the systematic medical record review, showing that 64 % of the GP reports for Lyme carditis met our criteria for very likely or likely diagnosis, and 38 % had a clinical presentation within the targeted period of 2009 and 2010. The adjusted incidence rate of six diagnoses of Lyme carditis per 10 million inhabitants in 2009 and 2010 may indicate a lower boundary of the true occurrence. Publications on underreporting of Lyme borreliosis suggest that the true incidence rate may be two to eight times higher than measured [8].

Diagnosing Lyme carditis can be very challenging, so our category of ‘very likely diagnosis’ still contains uncertainty. In the early stage of Lyme borreliosis serological assays for antibodies are infrequently positive, and antibody development can be inhibited or delayed by early antibiotic treatment for erythema migrans. Therefore, convalescent serological testing, some weeks after the acute phase, is sometimes required [1, 2]. In our criteria for classification (Table 1), we considered any positive serological finding relevant to assess in the context of clinical presentation, treatment and clinical course. Although conduction abnormalities, as well as *Borrelia*-specific IgG antibodies, are common among the general population, a coincidence of both is more likely to represent Lyme-related conduction abnormalities. In addition, other explanations for the cardiac condition need to be excluded [1, 2].

We consider the reported numbers of diagnoses and the medical records obtained from GPs representative of all cases diagnosed with Lyme carditis in the Netherlands, as we addressed the incidence questionnaire to all GPs nationwide, attaining a substantial response rate with a high coverage of our countries’ population. In our larger incidence survey on all manifestations of Lyme borreliosis in 2010 [6], we observed a response rate and incidence estimations of GP consultations for tick bites and erythema migrans that were similar to the preceding incidence surveys on 1994–2009 [4]. It is possible that willingness to cooperate in the medical record review might be higher among GPs who are more confident of the diagnosis. We are unable to quantify this potential response bias to the medical record review, which could influence our outcomes toward a higher proportion of likely diagnoses, and thus overestimation of the corrected incidence rate of Lyme carditis.

In line with our observation of 0.2 % diagnoses of Lyme carditis relative to all GP-reported clinical manifestations of Lyme borreliosis, other European physician-based studies report Lyme carditis in 0.3–0.7 % of cases with untreated Lyme borreliosis [9−11], and European epidemiological serology studies suggest that 0.3–4 % of patients with untreated Lyme borreliosis develop carditis [12−16]. So, Lyme carditis is diagnosed rarely relative to all manifestations of Lyme borreliosis. However, several European countries, including the Netherlands, have reported marked increases in the incidence of Lyme borreliosis [4, 5]. Failure to properly diagnose Lyme carditis may have serious consequences, with the possibility of sudden death or unnecessary implantation of a permanent pacemaker [3, 17]. According to the current study, approximately ten patients were diagnosed with Lyme carditis annually in the Netherlands in 2009 and 2010. This finding emphasises that physicians should include Lyme carditis into the differential diagnosis, in particular among younger individuals with cardiac symptoms in the presence of conduction abnormalities without other apparent risk factors, especially if combined with a history of recent exposure to ticks [1−3, 17].

## Conclusion

This study demonstrates that a nationwide representative incidence rate for Lyme carditis can be obtained rapidly by means of a two-step approach of a cross-sectional retrospective questionnaire among all GPs, followed by systematic review of medical records to adjust for misclassification and telescoping bias. We report the first incidence estimate for Lyme carditis in the Netherlands of six Lyme carditis cases per 10 million inhabitants annually, in 2009 and 2010. Although Lyme carditis remains a rare manifestation of Lyme borreliosis, physicians need to be aware of this diagnosis, in particular in countries where the incidence of Lyme borreliosis has increased during the past decades, as is the case in the Netherlands.
